# Crickets (*Acheta domesticus*) as Wheat Bread Ingredient: Influence on Bread Quality and Safety Characteristics

**DOI:** 10.3390/foods12020325

**Published:** 2023-01-09

**Authors:** Elena Bartkiene, Egle Zokaityte, Vytaute Starkute, Gintare Zokaityte, Aura Kaminskaite, Ernestas Mockus, Dovile Klupsaite, Darius Cernauskas, João Miguel Rocha, Fatih Özogul, Raquel P. F. Guiné

**Affiliations:** 1 Department of Food Safety and Quality, Veterinary Academy, Lithuanian University of Health Sciences, Tilzes Str. 18, LT-47181 Kaunas, Lithuania; 2 Institute of Animal Rearing Technologies, Faculty of Animal Sciences, Lithuanian University of Health Sciences, Tilzes Str. 18, LT-47181 Kaunas, Lithuania; 3 Food Institute, Kaunas University of Technology, Radvilenu Road 19, LT-50254 Kaunas, Lithuania; 4 LEPABE—Laboratory for Process Engineering, Environment, Biotechnology and Energy, Faculty of Engineering, University of Porto, Rua Dr. Roberto Frias, 4200-465 Porto, Portugal; 5 ALiCE—Associate Laboratory in Chemical Engineering, Faculty of Engineering, University of Porto, Rua Dr. Roberto Frias, 4200-465 Porto, Portugal; 6 Department of Seafood Processing Technology, Faculty of Fisheries, Cukurova University, 01330 Adana, Turkey; 7 CERNAS Research Centre, Polytechnic Institute of Viseu, 3504-510 Viseu, Portugal

**Keywords:** edible insects, wheat bread, fermentation, acrylamide, lactic acid bacteria

## Abstract

The aim of this study was to assess respondents’ opinions on the choice of edible insects as a food, and to evaluate the influence of cricket flour (ECF) (10, 20, 30%) on the quality of wheat bread (WB). Whereas ECF is an additional source of acrylamide precursors, in order to reduce acrylamide formation in WB, fermentation of ECF with *Lactiplantibacillus plantarum*-No.122 was applied. It was established that 70.7% of the respondents had never eaten insects and more than 30% would not choose them. However, ECF was suitable substrate for fermentation (lactobacilli count 8.24 log_10_CFU/g, pH-4.26). In addition, fermentation reduced the total biogenic amines content in ECF (by 13.1%). The highest specific volume showed WB, prepared with fermented ECF (10, 20, 30%). All the tested WB showed similar overall acceptability (on average, 7.9 points). However, the highest intensity of emotion “happy” was induced by the WB, prepared with fermented ECF. Most of the WB with non-treated and fermented ECF showed higher acrylamide concentration (except WB with 10% of fermented ECF), in comparison with the control. Finally, fermentation is recommended for ECF inclusion in the main WB formula because fermentation improves not only quality but also reduces acrylamide concentration in WB.

## 1. Introduction

The consumption of edible insects has a long tradition in certain countries [1]. Currently, especially in Western countries, insects are presented as prospective environmental and economic solutions for a more sustainable protein production [2]. It was reported that insects, as a part of the diet, can contribute to the growth of beneficial microorganisms as part of the gut microbiota [3]. They are also a good source of essential amino and fatty acids, fiber, vitamin B12, etc. [4]. For this reason, and in addition to their economic and environmental advantages, insects are highlighted as foods that provide health benefits [5]. The European Food Safety Authority (EFSA) recognized insects as novel foods through the Regulation (EU) 2015/2283, and the Food Agriculture Organization (FAO) recommended including them in Western diets [6,7].

The global insect market will steadily grow and the prognosis for the period 2020 to 2027 is that this market is expected to increase by 28.5% [8]. In addition, in the short-term, it is expected the increase in consumption of insects by 47% in Europe and North America [9]. However, the use of insects as food is still limited in the culture of Western countries because they are not a traditional diet [10]. One of the solutions to include insects in the main diet of the consumers in these countries may pass by be their incorporation into the main formulas of the traditional food, e.g., bread, as suggested as early as 1975 by Meyer–Rochow [11]. Insects are already used as ingredients in the production of bread, pasta, snacks, and meat substitutes, indeed [6,12,13,14].

Bread is a widely accepted and consumed food product throughout the world, and its consumption is growing [8]. Wheat bread (WB) provides a relatively high protein content of 11.48% [10]; however, it may lack certain essential amino acids. Moreover, in terms of consumers’ perception regarding insect-based food, their preferences fall on the consumption of traditional foodstuffs (bread, biscuits, bars, etc.) with the incorporation of insects, so that the insects are not visible and, simultaneously, the product provides high nutritional features and good sensory characteristics.

Cricket (*Acheta domesticus*) is a good source of protein (55–70%, dry matter) [15], its price is affordable, and its products are available all year round [16]. Cricket has good nutritional characteristics such as essential amino acids, unsaturated fatty acids, vitamins, minerals [5,6], chitin, and chitosan (desirable antimicrobial agents) [17]. The application of cricket flour in the food industry is very broad (pasta, snacks, bread, etc.), but the addition of proteinaceous materials to the food that is thermally treated can lead to an increase in the formation of acrylamide. The thermal treatment (including the baking process) improves product color, sensory properties, and texture; however, during this process, acrylamide may also be formed [18,19]. Acrylamide is a neurotoxic and carcinogenic compound present in foods with low moisture content and its formation is unleashed at temperatures higher than 120 °C [20]. In this regard, bread is placed in the risk group of food products because of its thermal treatment during baking, usually at temperatures higher than 220 °C. The indicative value for acrylamide in wheat bread (WB) and soft breads other than wheat-based ones are set at 80 and 150 μg/kg, respectively, according to scientific opinion on acrylamide in foods as reported by EFSA [21]. For acrylamide formation in bread, key factors are the water activity, fermentation (acidity, inoculated microorganisms, and, consequently, their metabolites), and baking (duration and temperature) [22]. Low water activity (a_w_) and high temperatures during thermal treatment are desired for specific crust properties’ formation, but these parameters lead simultaneously to a more intense Maillard reaction and, concomitantly, more acrylamide formation. The color and crispy texture of the bread crust are important parameters for the sensory properties of bread and consumer’s acceptability and, for this reason, solutions used for the purpose of acrylamide reduction in bread should not decrease the overall acceptability (OA) of the final product. Fermentation is an attractive biotechnology for the reduction of acrylamide in bread, because technological microorganisms (yeasts or sourdough lactic acid bacteria (LAB)) reduce acrylamide precursors in baking dough [23,24,25]. Asparagine is utilized during yeast fermentation [26], while sourdough LAB reduce acrylamide formation due to their amylolytic and proteolytic activities and ability to degrade acrylamide precursors; additionally, lower pH values of the dough obtained during sourdough or LAB fermentation lead to a lower acrylamide concentration in bread [27].

The aim of this research study was to assess respondents’ opinions about the perception of insects as a food or food ingredient and to evaluate the influence of cricket flour on the quality characteristics of wheat bread. Taking into consideration that cricket flour is an additional source of protein as well as of acrylamide precursors, the concentration of acrylamide in wheat bread was assessed. Intended to reduce acrylamide formation in wheat bread, fermentation with *Lactiplantibacillus plantarum* No. 122 (*Lp. plantarum* No. 122) strain was applied as a cricket flour pre-treatment. Considering that during fermentation both desirable and undesirable changes in the fermentable substrates can occur, additional analyses of the non-treated and fermented cricket flour were performed, viz.: pH, lactic acid bacteria viable counts, color coordinates, fatty acids (FA), volatile compounds (VC) and biogenic amines (BA). The tested wheat bread groups were prepared by adding to the main recipe 10, 20, and 30% of non-fermented (/non-treated) and fermented cricket flour, and bread samples were further subjected to analysis of specific volume, crumb porosity, shape coefficient, mass loss after baking, crust and crumb color coordinates, overall acceptability, and acrylamide concentration. In addition, induced emotions by the tested bread samples in a trained panel were evaluated.

## 2. Materials and Methods

### 2.1. Information about the Survey

This research was based on a questionnaire survey, using an instrument that was pre-validated for a sample of Portuguese participants [28,29] (Supplementary File S1. Questionnaire survey). This descriptive study was carried out on a non-probabilistic sample of 510 participants from Lithuania. All ethical principles were strictly followed when designing the questionnaire and data collection and treatment, namely those of the Declaration of Helsinki. The Ethics Committee approved this questionnaire survey with reference 45/SUB/2021. Data were collected between July and November 2021, using the electronic platform Google Forms, due to the restrictions caused by the COVID-19 pandemic. Only adult citizens (aged 18 years old or over) who expressed their informed consent were allowed to participate in the survey.

The participants’ age varied between 18 and 62 years old. Most participants were women (54.5%). Regarding the living environment, 63.9% lived in urban areas, with lower percentages living in suburban (31.6%) or rural areas (4.5%). With respect to education level, 31.2% were undergraduate, 38.6% completed a university degree and 30.2% had completed post-graduate studies (Master’s or doctoral degree).

### 2.2. Cricket Flour Fermentation and Breadmaking

The experimental design is summarized in Table 1.

#### 2.2.1. Cricket (*Acheta domesticus*) Flour and Lactic Acid Bacteria (LAB) Strain Used for Their Fermentation

Cricket (*Acheta domesticus*) flour was provided by Bugsandus Ltd. (Vilnius, Lithuania). Cricket flour composition was: protein 62.6%, fat 26.5%, ash 3.8%, moisture 2.0%, saturated fatty acids 8.7%, total carbohydrates 5.1%, sugars < 0.6%, and salt (sodium × 2.5) 0.69%.

The LAB strain *Lactiplantibacillus plantarum* No. 122 was acquired from the Lithuanian University of Health Sciences collection (Kaunas, Lithuania). Characteristics of the used LAB strain are reported by Bartkiene et al. [30].

#### 2.2.2. Cricket Flour Fermentation

Before the experiment, LAB strain was incubated and multiplied for 24 h in De Man, Rogosa, and Sharpe (MRS) broth culture medium (Biolife, Milano, Italy) at 30 °C under anaerobic conditions. A total of 3 mL of fresh LAB grown on MRS broth [average cell concentration of 8.6 log_10_ Colony-Forming Units (CFU)/mL] were inoculated in 100 g of cricket flour/water (ratio 1:1, *w*/*w*).

Afterwards, the cricket flour was fermented under anaerobic conditions in a chamber incubator (Memmert GmbH Co. KG, Schwabach, Germany) for 24 h at 30 °C. Non-fermented (/non-treated) cricket flour (mixed with water in the same mass ratio 1:1) was analyzed as a control.

Before and after fermentation, pH, color coordinates, LAB viable counts, biogenic amine (BA) concentration, and fatty acid and volatile compound profiles of the samples were analyzed.

#### 2.2.3. Analysis of pH, Color Characteristics, and Lactic Acid Bacteria (LAB) Viable Counts in the Cricket Flour and Total Color Change (ΔE) in the Cricket Flour and Bread

The pH of the samples was evaluated with a pH meter (Inolab 3, Hanna Instruments, Venet, Italy) by inserting the pH electrode into the cricket flour samples. The color coordinates of the cricket flour were evaluated on the surface using the CIE L*a*b* system (CromaMeter CR-400, Konica Minolta, Marunouchi, Tokyo, Japan).

Furthermore, the total color change (ΔE) between the control cricket flour/bread and the rest of the cricket flour/bread samples with 10, 20, and 30% of non-treated or fermented cricket flour was calculated using the equation according to Wrolstad and Smith [31]. The LAB viable counts were determined according to the method described by Bartkiene et al. [32].

#### 2.2.4. Analysis of Biogenic Amine (BA) Concentration in Cricket Flour

Sample preparation and determination of the biogenic amines (BA), including tryptamine, phenylethylamine, putrescine, cadaverine, histamine, tyramine, spermidine, and spermine, in cricket flour samples was conducted by following the procedure reported by Ben–Gigirey et al. [33] with some modifications described in Supplementary File S2.

#### 2.2.5. Analysis of Fatty Acid (FA) and Volatile Compound (VC) Profiles in the Cricket Flour

The extraction of lipids for fatty acid (FA) analysis was done with chloroform/methanol (2:1, *v*/*v*) and fatty acid methyl esters (FAME) were prepared according to Pérez-Palacios et al. [34]. All procedures are described in detail in Supplementary File S3.

The volatile compounds (VC) of the cricket flour samples were analyzed by gas chromatography–mass spectrometry (GC-MS). All procedures are described in detail in Supplementary File S3.

#### 2.2.6. Breadmaking and Quality Evaluation

The bread formula consisted of 1.0 kg of wheat flour, 1.5% salt, 3% instant yeast, and 1000 mL water (control bread). Control samples were prepared without the addition of non-fermented or fermented cricket flour. The tested bread groups were prepared by addition to the main recipe 10, 20, and 30% of non-fermented or fermented cricket flour. In total, seven groups of dough and bread samples were prepared, and the breads were tested (B_C_—control bread; B_CF10_; B_CF20_; B_CF30_—bread samples with 10, 20, and 30% of cricket flour, respectively; B_CF10-F122_; B_CF20-F122_; B_CF30-F122_—bread samples with 10, 20, and 30% of fermented cricket flour with *Lp. plantarum* No. 122, respectively). The baking dough was mixed for 2 min at a low speed, then for 6 min at a high-speed regime in a dough mixer (KitchenAid Artisan, OH, USA). Then, the baking dough was left at (24 ± 2) °C for 15 min relaxation. Afterwards, the dough was shaped into 375 g loaves, formed and proofed at (32 ± 2) °C and 80% relative humidity for 60 min. The bread was baked in a deck oven (EKA, Borgoricco PD, Italy) at 220 °C for 25 min.

After 12 h of cooling at (24 ± 2) °C, bread samples were subjected to the analyses of specific volume, crumb porosity, shape coefficient, mass loss after baking, crust and crumb color coordinates, overall acceptability, induced emotions for the trained judges, and acrylamide concentration.

Bread volume was established by American Association of Cereal Chemists (AACC) method [35], and the specific volume was calculated as the ratio of volume to weight. The bread shape coefficient was calculated as the ratio of bread slice width to height (in mm). Mass loss after baking was calculated as a percentage by measuring loaf dough mass before baking and after baking. Crust and crumb color parameters were evaluated using a CIE L*a*b* system (CromaMeter CR-400, Konica Minolta, Japan) [36]. Bread crumb hardness (after 24 and 48 h) was determined as the energy required for sample deformation (CT3 Texture Analyzer, Brookfield, WI, USA): bread slices of 2 cm thickness were compressed to 10% of their original height at a crosshead speed of 0.5 mm/s; the resulting peak energy of compression (mJ) was reported as crumb hardness. The moisture content was determined according to the International Association for Cereal Chemistry (ICC) standard method 110/1 [37]. Overall acceptability of breads was carried out by a trained panel of 10 judges using a 10-point Likert scale ranging from 10 (extremely like) to 0 (extremely dislike). Additional information is given in Supplementary File S4. Also, bread samples were tested by applying *FaceReader 8* software (Noldus Information Technology, Wageningen, The Netherlands), scaling the nine emotion patterns (neutral, happy, sad, angry, surprised, scared, disgusted, contempt and arousal) and valence. The judges were asked to taste the bread samples one by one in front of a Microsoft LifeCam Studio webcam (Microsoft Corporation, Redmond, WA, USA). The recordings were analyzed with *FaceReader 8* software and the intensity of facial expressions was expressed on a scale from 0 to 1, as well as valence from −1 to 1. Between the samples, the judges were asked to rinse the mouth with water.

Finally, the acrylamide concentration was determined according to the method of Zhang et al. [38] with modifications. All procedures are described in detail in Supplementary File S5.

### 2.3. Statistical Analysis

For the survey interpretation, basic descriptive statistics were used. For cricket flour and bread data interpretation, the results were expressed as the mean values ± standard error: for physicochemical parameters in cricket flour and bread samples, *n* = 3 ± standard error; and for bread overall acceptability and induced emotions with a trained panel, *n* = 10 ± standard error. In order to evaluate the effects of fermentation and different quantities of cricket flour on bread quality parameters, data were analyzed by one-way ANOVA and Tukey HSD tests as post hoc tests (statistical program R 3.2.1 (R Foundation for Statistical Computing, Vienna, Austria)). I addition, Pearson correlations were calculated between various parameters. The results were recognized as statistically significant at *p* ≤ 0.05.

## 3. Results and Discussion

### 3.1. Survey Results

**Figure 1:**
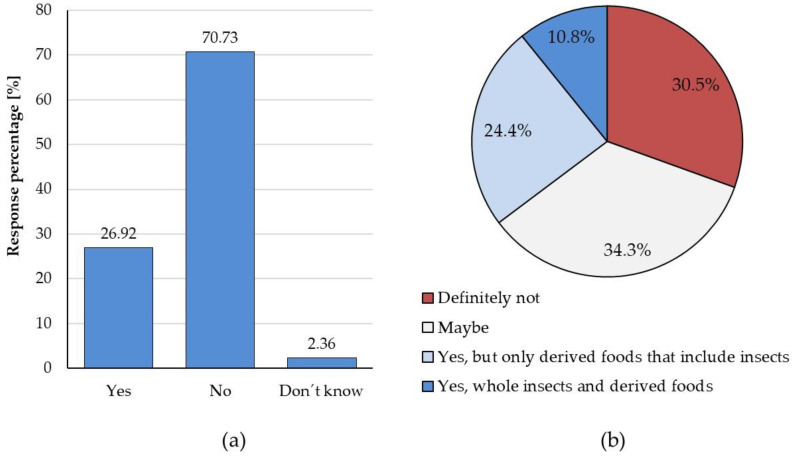
(**a**) Distribution of answers to the question “Have you ever eaten insects as culinary preparations, as snacks or other derived products?”. N = 510; (**b**) distribution of answers to the question “If you have never eaten insects, would you consider eating them?”

**Figure 2:**
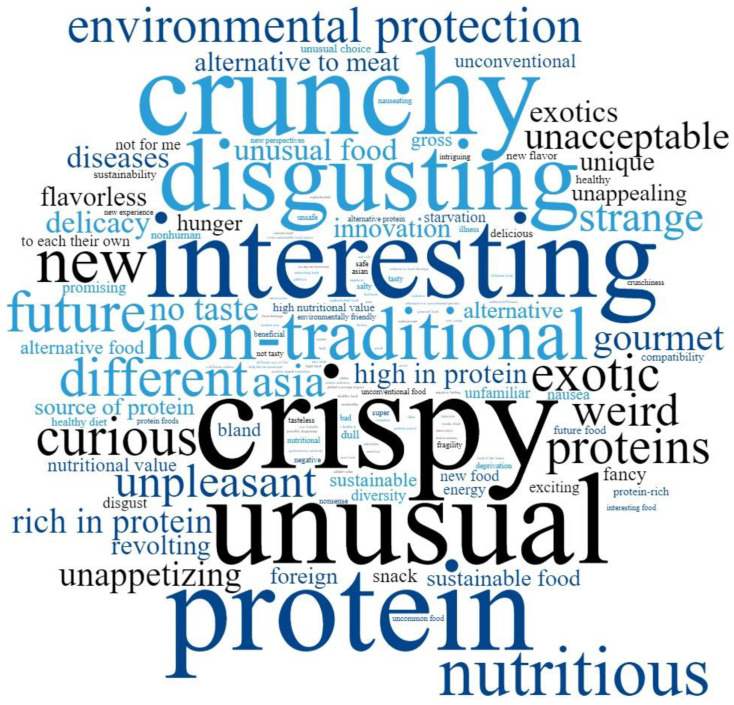
Word map of answers to the question “What comes to your mind when you hear about edible insects?”. Respondents were allowed to write up to 5 answers. Words are scaled by frequency.

Results of the survey showed that 70.7% of the respondents have never eaten insects as culinary preparation, snacks, or other derived products (Figure 1a). In addition, more than 30% would not consider eating insects (Figure 1b). Word map of answers to the question “What comes to your mind when you hear about edible insects?” showed that the most common words were “crispy”, “unusual”, “protein”, “interesting”, “crunchy”, and “disgusting” (Figure 2).

Most other studies have revealed that consumers are not very willing to eat insects [39]. Tuccillo et al. [40] reported that the consumption of insects was related to adventurous, daring, and wild emotions, while males were more positive and more willing to consume insects than females. The study of Reed et al. [41] showed that one-third of people in the USA are willing to try and eat products with insects regularly due to their taste and safety. Barton et al. [39] found that eating crickets was associated with being “disgusting” and “potentially harmful” for Western consumers. They also stated that in general the undesirable sensory properties of edible insects and the fear of eating them reduce the acceptance of edible insects. Moreover, in some cases, people who have consumed insects have a noticeably more favorable opinion of it [42].

### 3.2. Parameters of the Non-Treated and Fermented Cricket Flour

#### 3.2.1. Fermented and Non-Treated Cricket Flour pH, Color Coordinates, total Color Change (ΔE), and Lactic Acid Bacteria (LAB) Viable Counts

The pH, color coordinates, ΔE, and LAB viable counts of the cricket flour are given in Table 2. After 24 h of fermentation, cricket flour pH decreased, on average, 13.0%, and, after 48 h of fermentation, on average, 28.0%, in comparison with non-fermented one. Fermentation reduced cricket flour lightness (L*) and yellowness (b*) 32.0 and 24.8% on average, respectively, in comparison with non-treated samples. Fermentation also had a significant impact on the b* values of cricket flour (*p* = 0.035). According to the values of ΔE, the color of fermented cricket flour was different from the non-treated sample. Correlations between the pH and the color of the samples were not established. Moreover, fermentation was not significant on sample redness (a*). In fermented cricket flour, LAB viable count was 8.24 log_10_ CFU/g and there was a very strong negative correlation between samples’ pH and LAB viable counts (r = −0.836, *p* = 0.038).

The main desirable characteristics for microbial starter cultures (or simply starters) are the acidification rate and the effectiveness of microbial growth in the fermentable substrate [43]. It was reported that the *Lp. plantarum* CR L1 strain is suitable for cricket flour fermentation and bread preparation because of the good acidification rates and high LAB viable counts in sourdough [43,44]. However, it was reported that the highest LAB growth in cricket flour sourdough samples was established after 24 h of fermentation (9.01 log_10_ CFU/mL) [45]. In our study, the pH values after 24 h of cricket flour fermentation revealed that pH was not low enough for good quality sourdough characterization. The carbohydrates in fermentable substrate are a carbon source for the technological starters [46], while the compounds formed during the degradation of proteins are very important for LAB metabolism and its growth in the fermentable substrate [35]. The drop in the pH is associated with the production of organic acids, among others (for instance CO_2_); however, the insect flour can lead to a higher buffering capacity of the sourdough [43]. It was reported that the lactic, acetic, and succinic acids were the main organic acids produced during the insect flour fermentation with *Lp. plantarum* strain [45]. The latter strain showed a broad capacity for the metabolism of different phytochemicals [47].

#### 3.2.2. Biogenic Amine (BA) Concentration in Non-Treated and Fermented Cricket Flour

Biogenic amine (BA) concentrations in non-treated and fermented cricket flours are given in Table 3. The total BA content was lower in fermented samples (after 24 and 48 h of fermentation) when compared with non-fermented ones (on average 20.7 and 13.1% lower, respectively). However, when comparing individual BA concentrations, different tendencies were established. The highest tryptamine concentration was found in non-fermented samples (45.9 mg/kg), and in 24 and 48 h fermented samples, tryptamine content was on average 1.68 and 2.27 times lower, respectively. The lowest phenylethylamine content was found in 24 h fermented samples (20.7 mg/kg). However, by prolonging fermentation until 48 h, phenylethylamine concentration increased on average 1.97 times. In control (non-treated cricket flour) and 24 h fermented samples, putrescine concentration was on average 43.5 mg/kg, and after 48 h of fermentation, putrescine content was reduced on average by 2.86 times. In addition, in 48 h fermented samples cadaverine was not detected, and 24 h fermentation reduced cadaverine concentration in samples on average by 1.94 times. Histamine in both (non-treated and fermented) samples was not found; however, fermentation significantly increased tyramine concentration in cricket flour, *viz*.: after 24 h of fermentation on average by 13.0 times and after 48 h of fermentation on average by 18.9 times. A very strong negative correlation between tyramine concentration and samples pH values was observed (r = −0.824, *p* = 0.044).

The highest spermine and spermidine concentrations were found in control samples (on average 284.7 and 307.2 mg/kg, respectively). The latter BA concentrations in 24 and 48 h fermented samples were on average 144.3 and 167.0 mg/kg, respectively.

Studies about BA formation in cricket flour during fermentation are scarce. It was reported that the cricket powder hydrolysate by the *Yarrowia lipolytica* RO25 strain showed the lowest BA content among the analyzed samples [48]. For cricket flour, BA remains a hot topic of research due to its toxicological effects [49]. It was reported that the bread prepared with non-fermented cricket flour showed higher concentrations of cadaverine and tyramine, in comparison with bread prepared with cricket powder *Yarrowia lipolytica* RO25 hydrolysate [48]. The health hazards associated with consumption of crickets have not yet been deeply investigated [50]. Despite that the BA content in foods has been widely studied, the data about BA content in insect-based ingredients and products are scarce. Amino acid arginine can be converted to agmatine or to ornithine from which, during the decarboxylation pathway, putrescine is formed. The amino acid lysine can be decarboxylated into cadaverine. On the other hand, histamine, tyramine, tryptamine, and phenylethylamine can be formed from the amino acids histidine, tyrosine, tryptophan, and phenylalanine, respectively. Spermine is formed from spermidine, which is formed from putrescine by spermine and spermidine synthase, respectively [51]. In this study, a very strong positive correlation between spermine and spermidine concentrations was found (r = 0.999, *p* ≤ 0.0001). In addition, very strong positive correlations were established between putrescine and spermine, as well as between spermidine and putrescine (r = 0.940, *p* = 0.005 and r = 0.930, *p* = 0.007, respectively). Physiologically the most important BA are histamine and tyramine, because of their toxicity [52].

#### 3.2.3. Fatty Acid (FA) and Volatile Compound (VC) Profiles of Non-Treated and Fermented Cricket Flour

The fatty acid profile of non-treated and fermented cricket flour is tabulated in Table 4. The main fatty acids in cricket flour were methyl palmitate (C16:0), *cis,trans*-9- oleic acid methyl ester (C18:1 *cis,trans*), and methyl linoleate (C18:2).

It was reported that the fatty acid profile of crickets consists of oleic acids, linoleic acids, and palmitic acids, with small amounts of arachidonic acid [53]. Similar results were reported by Lucas et al., Skotnicka et al., and Wu et al. [54,55,56]. Additionally, controlled fermentation in insect flour led to an increase in saturated and monounsaturated fatty acids, while decreasing the content of polyunsaturated fatty acids [45].

Volatile compounds (only when the content of volatile compound was higher than 1% in at least one cricket flour sample are considered) of non-treated and fermented cricket flours are revealed in Table 5. Complementarily, volatile compounds whose content in cricket flour samples was below 1% are given in Supplementary File S6, Table S1. The main volatile compounds in non-treated cricket flour were acetic acid, hexanal, and decane (17.7, 15.7, and 28.1%, respectively, from the total content of volatile compounds). All of these compound concentrations decreased after fermentation and after 48 h of fermentation hexanal was absent in samples. Different trends of the 2-heptanone were found: after 24 h of fermentation the concentration of this compound was reduced on average 2.53 times; however, after 48 h of fermentation 2-heptanone content in cricket flour volatile compound profile increased on average 5.73 times. The highest content of 1-octen-3-ol, 2-pentylfuran, 4-methyldecane, 3,6-dimethyldecane, 3-methylundecane, ethyl octanoate, and dodecane was found in control samples and fermentation systematically reduced these volatile compounds content in cricket flour volatile compound profile. In opposite, acetoin, 2,3-butanediol, butanoic acid, 3-methylbutanoic acid, 2-methylbutanoic acid, 1-hexanol, 2,6-dimethylpyrazine, 2,2-dimethyl-3-heptanone, 2,6-dimethyl-4-heptanol, 2-hydroxy-3-methylpentanoic acid methyl ester, benzaldehyde, phenol, benzeneacetaldehyde, 3-ethyl-2,5-dimethylpyrazine, nonanal, and phenylethyl alcohol were detected just in fermented samples, showing the importance of lactic acid fermentation to develop flavors and aromas. Different tendencies of the tetramethylpyrazine, 2-nonanone, and 5-methylundecane were unfolded: the highest content of tetramethylpyrazine, and 2-nonanone was found in 48 h fermented samples; however, the highest content of 5-methylundecane was encountered in non-treated samples.

The *Lactobacillus plantarum* ATCC 8014 strain was mentioned as showing good properties for insect flour fermentation, including sourdough enrichment with fatty acids, amino acids, minerals, and volatile compounds (aldehydes, ketones, terpenes, and terpenoids, with pleasant odor perception such as benzaldehyde, 2-methyl-5-propan-2-ylcyclohex-2-en-1-one, *p*-cymene, and β-myrcene) [45]. Authors stated that the controlled insect fermentation led to the formation of 3-methyl-1-butanol (whiskey, malt, and burnt odor) and 2-methyl-5-propan-2-ylcyclohex-2-en-1-one (spicy, minty, caraway, bread, and rye bread odor), and that the spontaneous fermentation led to benzoic acid and disulfide dimethyl formation [45]. The odor of this volatile compound is described as unpleasant. Usually, volatile compound formation is described as amino acid reactions such as transamination, deamination, decarboxylation, and side chain modifications, which end with the development of alcohols, aldehydes, and acids [57]. In line with this, the development of benzaldehyde could be a result of the metabolic degradation of phenylalanine, whilst 3-methyl-1-butanol is considered one of the most frequently identified compounds and could derive from the leucine degradation [58]. Moreover, fatty acids can be precursors of volatile compound formation and their participation in volatile compound profile formation showed significant correlations between the separate volatile compounds and fatty acids (Supplementary File S6, Table S2).

### 3.3. Bread Quality and Safety Characteristics

#### 3.3.1. Influence of Cricket Flour on Bread Quality Characteristics

Bread-specific volume, porosity, shape coefficient, mass loss after baking, ΔE, and color characteristics are given in Table 6. The experimental data disclosed that by increasing non-fermented as well as fermented cricket flour content in the main bread formula, bread specific volume increased and the highest specific volume (on average 3.06 cm^3^/g) was found on breads prepared with fermented cricket flour (10, 20, and 30%). Similar tendencies were established regarding the bread porosity, that is the addition of non-treated as well as fermented cricket flour increased bread porosity in comparison with control breads (except bread prepared with 10% of non-treated cricket flour). However, the correlation between the specific volume and porosity of bread was not found. In both cases, fermented cricket flour showed better results in comparison with non-treated ones. Significant differences between shape coefficient, moisture content, and mass loss after baking the different groups of bread were not recognized. However, between bread porosity and bread moisture content or mass loss after baking, very strong positive correlations were found (r = 0.812, *p* ≤ 0.0001 and r = 0.988, *p* ≤ 0.0001, respectively). Comparing bread crust and crumb color coordinates, the addition of cricket flour (non-treated and fermented ones) to the main bread formula systematically reduced bread crust and crumb L*, increased crust and reduced crumb a*, and reduced crust and crumb b*. Comparing ΔE, it was found that the bread crust of B_CF10-F122_ was the most similar to the control bread crust (B_C_). Indeed, the total color change had proportionally risen from 21.05 to 31.51, and from 14.31 to 23.62, indicating significant differences between bread crust samples with 10% and 30% of non-treated and fermented cricket flour, respectively. It was found that the bread crumb of B_CF10_ was the most similar to the control bread crumb (B_C_). Moreover, ΔE had proportionally risen from 8.89 to 14.62, and from 11.56 to 14.42, indicating significant differences between bread crumb samples with 10% and 30% of non-treated or fermented cricket flour, respectively. Bread crust L* and b* coordinates showed positive correlations with bread porosity, moisture content, and mass loss after baking (for L*: r = 0.935 and *p* ≤ 0.0001, r = 0.859 and *p* ≤ 0.0001, and r = 0.937 and *p* ≤ 0.0001, respectively; and for b*: r = 0.949 and *p* ≤ 0.0001, r = 0.790 and *p* ≤ 0.0001, and r = 0.947 and *p* ≤ 0.0001, respectively). In addition, bread crumb L* showed positive correlations with bread porosity, moisture content, and mass loss after baking (r = 0.956 and *p* ≤ 0.0001, r = 0.824 and *p* ≤ 0.0001, r = 0.956 and *p* ≤ 0.0001, respectively), as well as bread crumb a* and b* showed positive correlations with bread porosity and mass loss after baking (for a*: r = 0.557 and *p* = 0.009, and r = 0.544 and *p* = 0.011, respectively; and for b*: r = 0.857 and *p* ≤ 0.0001, and r = 0.827 and *p* ≤ 0.0001, respectively). These results showed that not just the colored compounds of the cricket flour but also the changes that are obtained during the thermal treatment have a significant influence on the color parameters of the bread.

**Figure 3:**
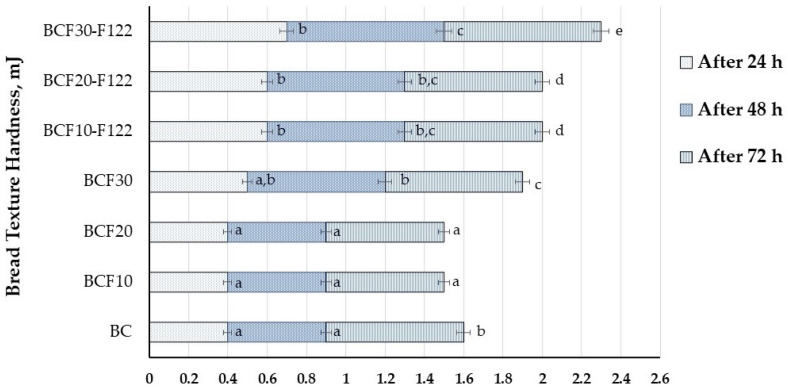
Bread texture hardness (mJ). B_C_—control bread; B_CF10_; B_CF20_; B_CF30_—bread samples with 10, 20, and 30% cricket flour, respectively; B_CF10-F122_; B_CF20-F122_; B_CF30-F122_—bread samples with 10, 20, and 30% cricket flour fermented with *Lactiplantibacillus plantarum* No. 122 strain, respectively. Data are expressed as mean values (*n* = 3) ± standard error (SE). ^a–e^ Mean values within a column with different letters are significantly different (*p* ≤ 0.05).

The changes in bread texture hardness during storage (after 24, 48, and 72 h) are shown in Figure 3. When comparing the hardness of bread samples after 24 h, significant differences between B_C,_ B_CF10_, and B_CF20_ samples were not found (on average bread hardness was 0.4 mJ). However, the addition of 30% of non-treated and 10, 20, and 30% of fermented cricket flour increased bread hardness to 0.5, 0.6, 0.6, and 0.7 mJ, respectively. After 48 h of storage samples with 30% fermented cricket flour showed the hardest texture (0.8 mJ). Similar tendencies remained after 72 h of storage; just the bread samples with 10 and 20% of non-treated cricket flour showed the lowest texture hardness (on average 0.6 mJ).

Breadmaking technology is sensitive to substituting wheat flour by gluten-free flour, owing to the decrease of the gluten network [59]. Moreover, the high ash content of the cricket powder can lead to a higher buffering capacity of the baking dough [43]. It was described that bread enrichment with cricket powder increased dough stability, reduced the degree of softening, and lowered water adsorption [6,13,44]. Conversely, it was reported that the inclusion of cricket powder to the main bread formula can reduce specific volume without decreasing the overall acceptability [43] and, as expected, bread volume decreases as the quantity of cricket flour substitution increases [6]. Cricket flour is rich in fat and Pareyt et al. stated that added fats led to the greater air penetration during the dough mixing process [60]. Certain lipid classes (for example diacylglycerols) integrated into the gluten network stabilize and strengthen the dough and during the thermal treatment (baking) the melting fat stabilizes the expanding gas cells. However, cricket flour was found to contribute to the poor gas retention capacity of the weakened gluten network [61]. These changes can be related to the decrease of gluten network, which causes inferior rheological performance of the baking dough [13,62]. Such differences reported by different authors can be related with different flour characteristics used for bread preparation, as well as different composition of insect flour.

One of the key-quality factor of bakery goods that affects consumer’s acceptability and preference is the color. It was reported that the color difference between control and insect flour-substituted breads was obvious to the human eye [63]. However, during baking there are several factors than have an influence on bread color: baking duration, temperature, chemical composition, physical properties of the treated product, etc. Maillard reaction takes place during baking between the amino groups and reducing sugars, which contribute to the observed differences in the content of individual color components in the crust and crumb of bread. The observed relationship between the reduction of the brightness of the bread crust and the substitution of wheat flour with insect flour is related, on one hand, to the darker color of insect flours when compared to wheat flour, and, on the other hand, it may be related to the protein content of these raw materials [53]. Finally, the portion of the raw material with a higher protein content contributed to the intensity of the Maillard reaction.

#### 3.3.2. Bread Overall Acceptability (OA) and Induced Emotions in Trained Judges

**Figure 4:**
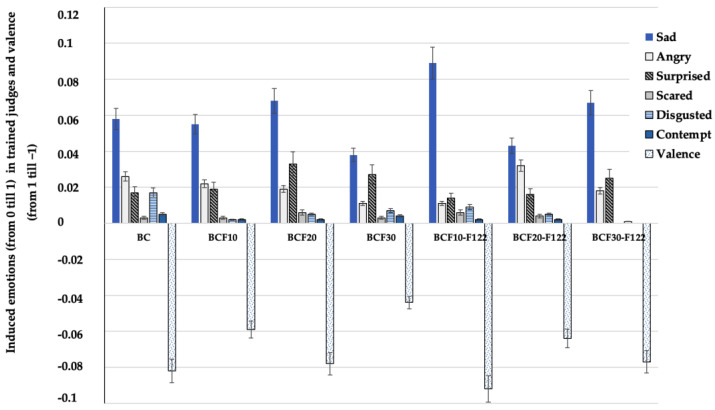
Intensity of induced emotions (“Sad”, “Angry”, Surprised”, “Scared”, “Disgusted”, “Contempt”) in trained judges and valence. B_C_—control bread; B_CF10_; B_CF20_; B_CF30_—bread samples with 10, 20, and 30% cricket flour, respectively; B_CF10-F122_; B_CF20-F122_; B_CF30-F122_—bread samples with 10, 20, and 30% cricket flour fermented with *Lactiplantibacillus plantarum* No. 122 strain, respectively. Data are expressed as mean values (*n* = 10) ± standard error (SE).

Results on the bread overall acceptability and induced emotions by bread samples in trained judges are shown in Table 7. Significant differences in the breads’ overall acceptability as well as induced emotion “neutral” between tested bread samples were not found. Furthermore, the emotion “neutral” induced by all the bread groups showed the highest intensity in comparison with other induced emotions in the trained judges. However, significant differences with the emotion “happy” were established, and the highest intensity of this emotion was induced by bread samples prepared with fermented cricket flour; moreover, by increasing their content in the main bread formula, the emotion “happy” intensity increased. Between the emotion “happy” and bread-specific volume a moderate positive correlation was found (r = 0.489, *p* = 0.024), as well between the emotion “happy” and bread crust a* a moderate negative correlation was recognized (r = −0.450, *p* = 0.041). Other emotions induced in trained judges by bread samples were lower than 0.100, as well as most of the valence of the samples was negative (Figure 4). Concerning negative emotions, the emotion with the highest intensity was “sad”, which ranged from 0.038 (in bread prepared with the addition of 30% of non-treated cricket flour) to 0.089 (in bread prepared with the addition of 10% of fermented cricket flour).

The emotions induced by various foods varied due to the person’s changing emotional state [64,65]. The emotions “happy” and “surprised” are more often associated with sweet foods than with salty, sour, or bitter ones, and a bitter taste is associated with the emotion “disgusted” [66,67,68]. However, new, unusual, non-traditional ingredients can induce different emotions. Despite it being reported that bread is associated with a “neutral” emotional status [67], new ingredients can induce other emotions. In this research study, the emotion “happy” was expressed more intensely for bread containing cricket flour. This could be associated with the new experience of testing a future protein source, unusual for Northeast European countries. Also, the judges were informed about the new ingredient, which could be associated with sustainable agriculture as well as with progress in reducing the problems associated with climate change.

#### 3.3.3. Acrylamide Content in Bread with Cricket Flour

Acrylamide concentration (µg/kg) in bread samples and bread crumb images are tabulated in Table 8. Most of the bread formulations with non-treated and fermented cricket flour showed higher acrylamide concentrations (except the bread samples prepared with 10% of fermented cricket flour), in comparison with control breads. In comparison acrylamide content in samples prepared with 10% of non-treated and 10% of fermented cricket flour, the latter group showed on average 33.2% lower acrylamide concentration. Samples prepared with 20% of non-treated and 20% of fermented cricket flour showed similar trends, and in samples prepared with fermented cricket flour, acrylamide was on average 31.7% lower, in comparison with samples prepared with non-treated ones. However, significant differences in acrylamide content, in samples prepared with 30% of non-treated and 30% of fermented cricket flour were not found, and acrylamide average concentration in these bread groups was 42.7 μg/kg.

It was stated that the addition of cricket flour to the main wheat bread formulation led to changes in the crust color, which could be related to a higher intensity of the Maillard reaction. As previously referred, the latter is associated with the formation of aroma and color compounds, as well as with a higher concentration of acrylamide in the end product. Indeed, the aspartic acid and asparagine content in cricket flour can reach 7.52% [44], and the latter amino acid is the main precursor for acrylamide formation. Moreover, it has been reported that the presence of lipids affects the Maillard reaction intensity [69]. In our study, correlations between the bread parameters and acrylamide content showed that the thermal treatment of bread (i.e., baking) and processes induced by the thermal treatment (not just Maillard reaction but also water migration from the dough) are very important for acrylamide formation (Table 9). The European Food Safety Authority (EFSA) reported that the possibility that other chemical compounds (including acrylamide) could be generated due to chemical reactions between insect compounds and other ingredients during processing should not be discarded [70]. Finally, the fermentation of cricket flour with the *Lactiplantibacillus plantarum* No. 122 strain can be recommended as a good mean to reduce acrylamide formation in bread samples.

## 4. Conclusions

In Lithuania insects are non-traditional food and results of the survey showed that more than 30% of the respondents do not want to choose them as food. For this reason, incorporation of the cricket flour into the main traditional food formulations becomes relatively attractive. However, additional proteinaceous ingredients in bread formula could lead to the higher formation of acrylamide. This study showed that the use of pre-treatment for cricket flour with selected LAB strain is an appropriate technology to reduce acrylamide formation in wheat bread enriched with cricket flour. Cricket flour was a suitable substrate for LAB fermentation: in 48 h fermented cricket flour LAB viable counts were 8.24 log_10_ CFU/g and pH 4.26. The total biogenic amine content in fermented cricket flour was 13.1% lower in comparison with non-fermented ones. The main fatty acids in cricket flour were methyl palmitate, *cis,trans*-9-oleic acid methyl ester, and methyl linoleate, and the main volatile compounds in non-treated cricket flour were acetic acid, hexanal, and decane. Fermented cricket flour (10, 20, and 30%) increased bread-specific volume. Significant differences between all bread samples in overall acceptability and the induced emotion “neutral” were not found. However, the highest intensity of the emotion “happy” was induced by the wheat bread prepared with fermented cricket flour. Most of the wheat bread formulations with non-treated and fermented cricket flour showed higher acrylamide concentrations, in comparison with the control bread samples. Finally, it can be stated that the inclusion of cricket flour in the main wheat bread formula leads to better quality characteristics of the baking product but, in most cases, increases acrylamide concentration (except in wheat bread prepared with 10% fermented cricket flour). According to the results obtained, fermentation of cricket flour before its use in wheat bread preparation is recommended as a pre-treatment because fermentation increases not only the quality but also the safety characteristics of wheat bread.

## Figures and Tables

**Table 1:** Representation of the experimental design with definition of the factors and observations.

Independent Variables/Factors	Dependent Variables/Observations
CRICKET FLOUR SAMPLES	
Non-fermented (/non-treated/without pre-treatment) cricket flour [100 g of cricket flour/water, ratio 1:1 (*w*/*w*)] (control cricket flour) (CoCr)	pH Lactic acid bacteria (LAB) viable counts Color coordinates Total color change (ΔE) Fatty acids (FA) Volatile compounds (VC) Biogenic amines (BA)
Fermented cricket flours (100 g of cricket flour/water, ratio 1:1 (*w*/*w*)) for 24 and 48 h (Cr_122_)	
WHEAT BREAD SAMPLES WITH CRICKET FLOUR	
Wheat bread (control bread) (Bc) [1.0 kg of wheat flour, 1.5% salt, 3% instant yeast and 1000 mL water]	Specific volume Crumb porosity Shape coefficient Mass loss after baking Crust and crumb color coordinates Total color change (ΔE) Overall acceptability (OA) Induced emotions Acrylamide concentration
Wheat bread with 10% of non-fermented cricket flour (B_CF10_)	
Wheat bread with 20% of non-fermented cricket flour (B_CF20_)	
Wheat bread with 30% of non-fermented cricket flour (B_CF30_)	
Wheat bread with 10% of fermented cricket flour (B_CF10-F122_)	
Wheat bread with 20% of fermented cricket flour (B_CF20-F122_)	
Wheat bread with 30% of fermented cricket flour (B_CF30-F122_)	

**Table 2:** pH, color coordinates, total color change (ΔE), and lactic acid bacteria (LAB) viable counts in non-treated and fermented cricket flours.

Samples	pH			Colour Coordinates, NBS			ΔE	LAB Count, log_10_ CFU/g (after 48 h)
	Duration of Fermentation, h			L*	a*	b*		
	0	24	48					
CoCr	5.94 ± 0.43 a	na	na	53.4 ± 1.9 b	2.25 ± 0.13 a	15.7 ± 1.9 b	na	na
Cr_122_	5.92 ± 0.55 a	5.15 ± 0.03	4.26 ± 0.04	36.3 ± 1.12 a	2.12 ± 0.17 a	11.8 ± 1.0 a	17.54 ± 1.93	8.24 ± 1.21

Co—control; Cr—cricket flour; 122—cricket flour fermented with *Lactiplantibacillus plantarum* No. 122 strain. L* lightness; a* redness; b* yellowness; NBS—National Bureau of Standards units; ΔE—total color change. Data are expressed as mean values (*n* = 3) ± SE; SE—standard error; na—not analysed. a–b—Mean values between samples within a column with different letters are significantly different (*p* ≤ 0.05).

**Table 3:** Biogenic amine (BA) concentration (mg/kg) in non-treated and fermented cricket flours.

Biogenic Amines, mg/kg	Cricket Flour		
	CoCr	Cr_122_	
	Duration of Fermentation, h		
	0	24	48
Tryptamine (TRP)	45.9 ± 4.15 c	27.3 ± 3.55 b	20.2 ± 1.39 a
Phenylethylamine (PHE)	30.2 ± 2.25 b	20.7 ± 2.25 a	40.8 ± 2.85 c
Putrescine (PUT)	40.8 ± 3.08 b	46.2 ± 7.66 b	15.2 ± 1.38 a
Cadaverine (CAD)	69.3 ± 4.76 b	35.8 ± 3.91 a	nd
Histamine (HIS)	nd	nd	nd
Tyramine (TYR)	15.4 ± 1.70 a	199.6 ± 15.2 b	291.0 ± 21.5 c
Spermidine (SPRMD)	284.7 ± 27.61 b	138.3 ± 11.5 a	150.3 ± 13.7 a
Spermine (SPRM)	307.2 ± 21.84 b	161.7 ± 14.6 a	172.2 ± 14.4 a

Co—control; Cr—cricket flour; 122—cricket flour fermented with *Lactiplantibacillus plantarum* No. 122 strain. Data are expressed as mean values (*n* = 3) ± SE; SE—standard error; nd—not detected. a–c Mean values between samples within a column with different letters are significantly different (*p* ≤ 0.05). TRP—tryptamine; PHE—phenylethylamine; CAD—cadaverine; HIS—histamine; TYR—tyramine; SPRMD—spermidine; SPRM—spermine.

**Table 4:** Fatty acid (FA) composition (% from the total fat content) in non-treated and fermented cricket flours.

Fatty Acid Composition, % from the Total Fat Content	Cricket Flour		
	CoCr	Cr_122_	
	Duration of Fermentation, h		
	0	24	48
C16:0	26.3 ± 0.11 b	26.8 ± 0.13 c	24.6 ± 0.11 a
C16:1	0.138 ± 0.00 6	nd	nd
C18:0	8.72 ± 0.12 c	8.40 ± 0.05 b	7.81 ± 0.12 a
C18:1 *cis*, *trans*	27.4 ± 0.14 a	28.9 ± 0.12 b	30.5± 0.3 c
C18:2	35.7 ± 0.16 b	33.8 ± 0.14 a	35.2 ± 0.4 b
C18:3 α	1.52 ± 0.03 a	2.00 ± 0.06 b	1.92 ± 0.04 b

Co—control; Cr—cricket flour; 122—cricket flour fermented with *Lactiplantibacillus plantarum* No. 122 strain; nd—not determined. Data are expressed as mean values (*n* = 3) ± SE; SE—standard error. a–c—Mean values between samples within a row with different letters are significantly different (*p* ≤ 0.05). C16:0—methyl palmitate; C16:1—methyl palmitoleate; C18:0—methyl stearate; C18:1 *cis,trans—cis,trans*-9-oleic acid methyl ester; C18:2—methyl linoleate; C18:3 α—α-linolenic acid methyl ester.

**Table 5:** Volatile compounds in non-treated and fermented cricket flours (only volatile compounds whose content in the cricket flour samples was in at least one sample >1% are considered).

RT, min	Volatile Compounds	Cricket Flour		
		CoCr	Cr_122_	
		Duration of Fermentation, h		
		0	24	48
2.315	Acetic acid	17.7 ± 2.40 b	6.82 ± 1.10 a	7.76 ± 1.19 a
3.754	Acetoin	nd	28.3 ± 5.44	nd
5.435	2,3-Butanediol	nd	1.40 ± 0.190	nd
5.926	Hexanal	15.7 ± 2.31 b	3.46 ± 0.604 a	nd
6.171	Butanoic acid	nd	nd	0.766 ± 0.081
7.861	3-methylbutanoic acid	nd	23.5 ± 4.53 b	7.52 ± 0.056 a
8.048	2-methylbutanoic acid	nd	1.88± 0.185 b	1.11 ± 0.119 a
8.089	1-Hexanol	nd	4.09 ± 0.914	nd
8.765	2-Heptanone	6.18 ± 1.05 b	2.44 ± 0.494 a	35.4 ± 4.82 c
9.342	2,6-dimethylpyrazine	nd	0.310 ± 0.036 a	3.29 ± 0.452 b
10.512	2,2-Dimethyl-3-heptanone	nd	0.974 ± 0.137 a	0.817 ± 0.164 a
10.683	2,6-dimethyl-4-heptanol	nd	3.20 ± 0.408 b	1.17 ± 0.127 a
10.93	2-hydroxy-3-methylpentanoic acid methyl ester	nd	0.506 ± 0.071 a	0.532 ± 0.099 a
11.12	Benzaldehyde	nd	1.09 ± 0.106 a	1.17 ± 0.240 a
11.755	1-Octen-3-ol	2.29 ± 0.161 b	0.311 ± 0.051 a	0.388 ± 0.033 a
11.88	Phenol	nd	nd	1.53 ± 0.143 a
12.207	2-pentylfuran	2.95 ± 0.352 b	1.43 ± 0.184 a	1.52 ± 0.115 a
12.493	Decane	28.1 ± 2.64 c	7.34 ± 0.777 a	13.29 ± 1.28 b
13.269	4-methyldecane	1.62 ± 0.313 c	0.511 ± 0.058 b	0.420 ± 0.034 a
13.896	Benzeneacetaldehyde	nd	0.577 ± 0.088 a	0.932 ± 0.083 b
14.42	3,6-dimethyldecane	2.92 ± 0.284 c	0.945 ± 0.075 a	1.36 ± 0.140 b
15.071	3-ethyl-2,5-dimethylpyrazine	nd	0.519 ± 0.044 a	1.45 ± 0.132 b
15.313	Tetramethylpyrazine	0.928 ± 0.093 b	0.405 ± 0.032 a	1.64 ± 0.141 c
15.518	2-Nonanone	1.55 ± 0.197 b	0.419 ± 0.037 a	6.18 ± 0.557 c
15.886	5-methylundecane	1.42 ± 0.247 b	nd	0.554 ± 0.056 a
15.94	Nonanal	nd	0.596 ± 0.052	nd
16.264	Phenylethyl Alcohol	nd	1.41 ± 0.270	nd
18.081	3-methylundecane	1.26 ± 0.114 c	0.323 ± 0.033 b	0.208 ± 0.040 a
18.909	Ethyl octanoate	1.43 ± 0.133 c	0.629 ± 0.069 b	0.573 ± 0.022 a
18.967	Dodecane	9.75 ± 0.88 c	2.87 ± 0.221 a	4.09 ± 0.075 b

Co—control; Cr—cricket flour; 122—cricket flour fermented with *Lactiplantibacillus plantarum* No. 122 strain; nd—not determined. Data are expressed as mean values (*n* = 3) ± SE; SE—standard error. a–c—Mean values between samples within a row with different letters are significantly different (*p* ≤ 0.05).

**Table 6:** Bread specific volume, porosity, shape coefficient, mass loss after baking, total color change (ΔE), and color characteristics of the bread crust and crumb.

Bread Samples	Specific Volume, cm^3^/g		Shape Coefficient		Porosity, %	Moisture Content, %		Mass Loss after Baking, %
B_C_	2.31 ± 0.23 a		1.82 ± 0.12 a		62.8 ± 1.36 a	21.5 ± 1.14 a		11.3 ± 1.05 a
B_CF10_	2.43 ± 0.15 a,b		1.91 ± 0.11 a		64.4 ± 2.36 a	22.6 ± 0.98 a		11.6 ± 1.21 a
B_CF20_	2.56 ± 0.18 b,c		1.93 ± 0.08 a		65.6 ± 2.55 a,b	22.4 ± 1.02 a		11.8 ± 1.13 a
B_CF30_	2.71 ± 0.21 c		1.96 ± 0.11 a		68.3 ± 1.98 b	22.8 ± 1.30 a		12.0 ± 1.11 a
B_CF10-F122_	2.88 ± 0.24 c,d		2.01 ± 0.13 a		70.3 ± 3.61 b	21.9 ± 1.25 a		12.5 ± 1.36 a
B_CF20-F122_	3.05 ± 0.29 c,d		2.05 ± 0.15 a		73.5 ± 2.87 b,c	21.6 ± 1.56 a		12.4 ± 1.23 a
B_CF30-F122_	3.26 ± 0.30 d		2.03 ± 0.10 a		75.3 ± 1.59 c	21.7 ± 1.36 a		11.9 ± 1.14 a
	Color coordinates, NBS							
Bread samples	Crust				Crumb			
	L*	a*	b*	ΔE	L*	a*	b*	ΔE
B_C_	81.0 ± 0.23 e	0.513 ± 0.026 a	19.4 ± 0.13 f	na	58.9 ± 0.32 e	13.05 ± 0.11 f	21.0 ± 0.20 e	na
B_CF10_	60.8 ± 0.54 c	2.97 ± 0.14 e	14.0 ± 0.12 c	21.05 ± 0.21 b	51.4 ± 0.41 d	8.86 ± 0.19 a	18.7 ± 0.17 c	8.89 ± 0.16 a
B_CF20_	55.2 ± 0.32 a	2.53 ± 0.32 d,e	12.9 ± 0.11 a	26.68 ± 0.18 c	45.1 ± 0.23 b	10.9 ± 0.15 b	16.0 ± 0.15 b	14.83 ± 0.11 c
B_CF30_	49.9 ± 0.26 a	2.81 ± 0.15 e	14.9 ± 0.13 d	31.51 ± 0.14 d	45.4 ± 0.39 b	12.6 ± 0.13 e	15.4 ± 0.14 a	14.62 ± 0.14 c
B_CF10-F122_	67.1 ± 0.41 d	1.72 ± 0.11 c	16.2 ± 0.14 e	14.31 ± 0.22 a	47.7 ± 0.28 c	11.1 ± 0.10 c	18.9 ± 0.17 c	11.56 ± 0.10 b
B_CF20-F122_	58.2 ± 0.28 b	1.49 ± 0.09 b	14.2 ± 0.13 c	23.41 ± 0.19 b	44.6 ± 0.31 a	12.0 ± 0.14 d	20.1 ± 0.19 d	14.37 ± 0.16 c
B_CF30-F122_	58.1 ± 0.39 b	2.32 ± 0.16 d	13.9 ± 0.12 b	23.62 ± 0.20 b	44.5 ± 0.42 a	12.6 ± 0.16 e	20.5 ± 0.18 d	14.42 ± 0.13 c

Data are expressed as mean values (*n* = 3) ± standard error (SE). a–f—Mean values within a row with different letters are significantly different (*p* ≤ 0.05); na—not analyzed. L* lightness; a* redness; b* yellowness; NBS—National Bureau of Standards units; ΔE—total color change. B_C_—control bread; B_CF10_; B_CF20_; B_CF30_—bread samples with 10, 20, and 30% cricket flour, respectively; B_CF10-F122_; B_CF20-F122_; B_CF30-F122_—bread samples with 10, 20, and 30% cricket flour fermented with *Lactiplantibacillus plantarum* No. 122 strain, respectively.

**Table 7:** Bread overall acceptability (OA) and induced emotions in trained judges.

Emotions and OA	Bread Samples						
	B_C_	B_CF10_	B_CF20_	B_CF30_	B_CF10-F122_	B_CF20-F122_	B_CF30-F122_
Neutral	0.839 ± 0.085 a	0.789 ± 0.079 a	0.837 ± 0.081 a	0.870 ± 0.088 a	0.830 ± 0.084 a	0.782 ± 0.079 a	0.856 ± 0.086 a
Happy	0.082 ± 0.009 b	0.044 ± 0.005 a	0.053 ± 0.006 c	0.173 ± 0.016 d	0.291 ± 0.030 e	0.264 ± 0.029 e	0.432 ± 0.052 f
OA	8.00 ± 1.95 a	8.03 ± 1.90 a	7.13 ± 2.56 a	8.38 ± 1.61 a	7.50 ± 2.30 a	7.88 ± 2.11 a	8.38 ± 1.50 a

OA—overall acceptability; B_C_—control bread; B_CF10_; B_CF20_; B_CF30_—bread samples with 10, 20 and 30% cricket flour, respectively; B_CF10-F122_; B_CF20-F122_; B_CF30-F122_—bread samples with 10, 20 and 30% cricket flour fermented with *Lactiplantibacillus plantarum* No. 122 strain, respectively. Data are expressed as mean values (*n* = 10) ± standard error (SE). a–f—Mean values within a row with different letters are significantly different (*p* ≤ 0.05).

**Table 8:** Acrylamide concentration (µg/kg) in bread samples and bread crumb images.

Bread Samples	Acrylamide Concentration µg/kg	Image
B_C_	21.2 ± 1.92 a	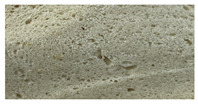
B_CF10_	37.6 ± 2.12 c	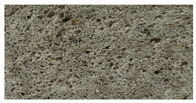
B_CF20_	45.4 ± 3.14 d	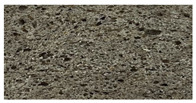
B_CF30_	43.1 ± 3.25 d	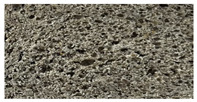
B_CF10-F122_	25.1 ± 1.98 a	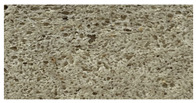
B_CF20-F122_	31.0 ± 1.18 b	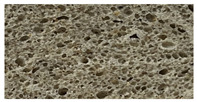
B_CF30-F122_	42.3 ± 3.28 d	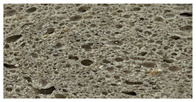

B_C_—control bread; B_CF10_; B_CF20_; B_CF30_—bread samples with 10, 20, 30% cricket flour, respectively; B_CF10-F122_; B_CF20-F122_; B_CF30-F122_—bread samples with 10, 20, and 30% cricket flour fermented with *Lactiplantibacillus plantarum* No. 122 strain, respectively. Data are expressed as mean values (*n* = 3) ± standard error (SE). a–d—Mean values within a line with different letters are significantly different (*p* ≤ 0.05).

**Table 9:** Correlation between acrylamide concentration in bread samples and bread parameters.

Bread Parameters	Pearson’s Correlation between Acrylamide Content and Analysed Bread Parameters	*p*
Bread porosity	0.903 **	0.0001
Bread moisture content	0.655 **	0.001
Bread mass loss after baking	0.897 **	0.0001
Crust L*	0.760 **	0.0001
Crust b*	0.782 **	0.0001
Crumb L*	0.825 **	0.0001
Crust a*	0.606 **	0.004
Crumb b*	0.854 **	0.0001

L* lightness; a* redness; b* yellowness; *p*—significance. * Correlation is significant at the 0.05 level (2-tailed). ** Correlation is significant at the 0.01 level (2-tailed).

## Data Availability

Data are available from the corresponding author upon request.

## Supplementary Materials

The following supporting information can be downloaded at: https://www.mdpi.com/article/10.3390/foods12020325/s1, Supplementary File S1. Questionnaire survey; Supplementary File S2. Analysis of biogenic amine concentration in edible cricket flour; Supplementary File S3. Analysis of fatty acid and volatile compound profiles; Supplementary File S4. Sensory analysis of bread with cricket flour [71,72,73,74,75,76]; Supplementary File S5. Analysis of acrylamide concentration in bread; Supplementary File S6. Edible cricket flour volatile compounds.

## Abbreviations

α-Linolenic acid methyl ester (C18:3 α), American Association of Cereal Chemists (AACC), biogenic amines (BA), blueness (-b*), cadaverine (CAD), *cis,trans*-9- oleic acid methyl ester (C18:1 *cis,trans*), colony-forming units (CFU), De Man, Rogosa, and Sharpe (MRS), edible cricket flour (ECF), European Food Safety Authority (EFSA), European Food Safety Authority (EFSA), fatty acids (FA), fatty acids methyl esters (FAME), Food Agriculture Organization (FAO), gas chromatography-mass spectrometry (GC-MS), greenness (-a*), histamine (HIS), International Association for Cereal Chemistry (ICC), lactic acid bacteria (LAB), *Lactiplantibacillus plantarum* (*Lp. plantarum*), lightness (L*), methyl linoleate (C18:2), methyl palmitate (C16:0), methyl palmitoleate (C16:1), methyl stearate (C18:0), National Bureau of Standards (NBS), overall acceptability (OA), phenylethylamine (PHE), putrescine (PUT), redness (a*), spermidine (SPRMD), spermine (SPRM), standard error (SE), total color change (ΔE), tryptamine (TRP), tyramine (TYR), volatile compounds (VC), water activity (a_w_), wheat bread (WB), yellowness (b*).
