# Supplementary material for: Crickets (Acheta domesticus) as Wheat Bread Ingredient: Influence on Bread Quality and Safety Characteristics
Source: Foods. 2023 Jan 9;12(2):325. doi: 10.3390/foods12020325 (PMC9858247; doi:10.3390/foods12020325)
Supplement: Supplementary file 1 [file foods-12-00325-s001.zip › Supplementary File S1. Questionnaire survey.pdf]

EDIBLE INSECTS SURVEY

This data collection is intended to investigate consumer perspectives and knowledge about insects for human consumption.

It is developed within the scope of the EISuFood project taking place in 18 countries simultaneously (Coordinator: Raquel Guiné, Portugal & Elena Bartkiene, Lithuania).

Ethical principles are strictly followed, participation is voluntary and all information collected is strictly confidential. Only adult participants, who give consent, respond to the questionnaire.

Thank you in advance for your cooperation.

We thank you in advance for your collaboration.

☐ I am 18 years old or over and I agree to participate in the survey.

1. Demographic Data

1. **Age:** _____ years

2. **Sex:**

Female ☐ 1 Male ☐ 2 Don't want to answer ☐ 3

3. **Education level:**

Post-graduate education (master or doctorate) ☐ 1

Completed a university degree ☐ 2

No university degree ☐ 3

If you do not have a university degree, how many school years you have:____________ 3.a

4. **Living environment:**

Rural ☐ 1 Urban ☐ 2 Suburban ☐ 3

2. Characterization of participants' habits

5. Have you ever eaten insects as culinary preparations, as snacks or other derived products?

Yes ☐ 1 No ☐ 2 Don´t know/Don´t remember ☐ 3

6. If you have never eaten insects, would you consider eating them?

Definitely not ☐ 1 Maybe ☐ 2
Yes, but only derived foods that include insects (for example hamburger or biscuits) ☐ 3
Yes, whole insects and derived foods ☐ 4

7. What comes to your mind when you hear about edible insects? Please use up to 5 words or small expressions that you associate with edible insects.

1)____

2)____

3)____

4)____

5)____
