# Supplementary material for: Crickets (Acheta domesticus) as Wheat Bread Ingredient: Influence on Bread Quality and Safety Characteristics
Source: Foods. 2023 Jan 9;12(2):325. doi: 10.3390/foods12020325 (PMC9858247; doi:10.3390/foods12020325)
Supplement: Supplementary file 1 [file foods-12-00325-s001.zip › Supplementary File S2. Analysis of biogenic amine concentration in edible cricket flour.pdf]

Analysis of biogenic amine concentration in edible cricket flour

Sample preparation and determination of the biogenic amines (BAs), including tryptamine, phenylethylamine, putrescine, cadaverine, histamine, tyramine, spermidine, and spermine in samples was conducted by following the procedure reported by Ben-Gigirey et al. [19] with some modifications described below.

Briefly, the standard BA solutions were prepared by dissolving known amounts of each BAs (including internal standard) in 20 mL of deionized water. The extraction of BAs in samples (5 g) was done by using 0.4 mol/L perchloric acid. The derivatization of sample extracts and standards was performed using a dansyl chloride solution (10 mg/mL) as a reagent.

The chromatographic analyses were carried out using a Varian ProStar HPLC system (Varian Corp., Palo Alto, California, USA) with two ProStar 210 pumps, a ProStar 410 auto-sampler, a ProStar 325 UV/VIS Detector and Galaxy software (Agilent, Santa Clara, California, USA) for data processing. For the separation of amines, a Discovery ® HS C18 column (150 × 4.6 mm, 5 µm; SupelcoTM Analytical, Bellefonte, Pennsylvania, USA) was used.

The eluents were ammonium acetate (A) and acetonitrile (B) and the elution program consisted of a gradient system with a 0.8 mL/min flow-rate. The duration of the analysis was 28 minutes. The eluent composition changed from 50% B to 90% B (respectively, from 50% A to 10% A) for the first 17 minutes, then remained constant at 90% B (10% A) for 3 minutes. Later, to ensure the separation of substances for the next analysis, the column was filled with eluent, whose composition is 50% B and 50% A, for 8 minutes. The detection wavelength was set to 254 nm, the oven temperature was 40 °C and samples were injected in 20 µL aliquots.

The target compounds were identified based on their retention times in comparison to their corresponding standards.
