# Supplementary material for: Crickets (Acheta domesticus) as Wheat Bread Ingredient: Influence on Bread Quality and Safety Characteristics
Source: Foods. 2023 Jan 9;12(2):325. doi: 10.3390/foods12020325 (PMC9858247; doi:10.3390/foods12020325)
Supplement: Supplementary file 1 [file foods-12-00325-s001.zip › Supplementary File S3. Analysis of fatty acid and volatile compound profiles.pdf]

Analysis of fatty acid profile of the cricket flour samples

The extraction of lipids for fatty acid (FA) analysis was done with chloroform/methanol (2:1 v/v) and FA methyl esters (FAME) were prepared according to Pérez-Palacios et al. [20].

The FA composition of the edible cricket flour samples was identified using a gas chromatograph GC - 2010 Plus (Shimadzu Europa GmbH, Duisburg, Germany) equipped with Mass Spectrometer GCMS-QP2010 (Shimadzu Europa GmbH, Duisburg, Germany).

Separation was carried out on a Stabilwax-MS column (30 m length, 0.25 mmID, and 0.25 µm df) (Restek Corporation, Bellefonte, US). Mass spectrometer operated at full scan mode. Analyte was injected in split mode at 1:60 split ratio. The following parameters were used: MS ion source temperature: 240°C, MS interface temperature 240°C, helium (carrier gas) flow: 0.90 mL/min, injector: 240°C, oven temperature 50°C (4 min), 10°C/min to 110°C (1 min), 15°C/min to 160°C (2 min), 2.5°C/min to 195°C (1 min), 2°C/min to 230°C (1 min), 2°C/min to 240°C (12 min).

The individual FAME peaks were identified by comparing their retention times with FAME standards (Merck & Co., Inc., Kenilworth, NJ, USA). Composition was evaluated using corrected area normalization method.

Analysis of volatile compound profile of the cricket flour

The volatile compounds (VC) of the edible cricket flour samples were analysed by gas chromatography-mass spectrometry (GC-MS).

A solid phase microextraction (SPME) device with Stableflex™ fibre coated with a 50 µm PDMS-DVB-Carboxen™ layer (Supelco, USA) was used for analysis. For headspace extraction, 1 g of sample and 10 mL of 1M phosphate buffer (pH=3) were transferred to the 20 mL extraction vial, mixed, sealed with a polytetrafluoroethylene septum, and thermostatted at 60°C for 30 min before exposing the fibre in the headspace.

The fibre was exposed to the headspace of the vial for 10 min and desorbed in an injector liner for 2 min (splitless injection mode). Prepared samples were analysed with a GCMS-QP2010 (Shimadzu, Japan) gas chromatograph and mass spectrometer.

The following conditions were used for analysis: injector temperature 250°C, ion source temperature 220°C and interface temperature 260°C. Helium was used as a carrier gas at 0.65 mL/min flowrate. For separation of VC, a low polarity Rxi®-5MS column (Restek, USA) (length 30 m, coating thickness 0.25 µm, inner diameter of 0.25 mm) was used. The temperature gradient was programmed from starting at 40°C (3 min hold) to 220°C (5°C/min) up to 310°C (15°/min) (6 min hold).

The VC were identified according to mass spectrum libraries (NIST11, NIST11S, FFNSC2). For identification purposes, alkane mix (C8-C20) were analyzed to obtain the retention indexes of unknown compounds.
