# Supplementary material for: Crickets (Acheta domesticus) as Wheat Bread Ingredient: Influence on Bread Quality and Safety Characteristics
Source: Foods. 2023 Jan 9;12(2):325. doi: 10.3390/foods12020325 (PMC9858247; doi:10.3390/foods12020325)
Supplement: Supplementary file 1 [file foods-12-00325-s001.zip › Supplementary File S4. Sensory analysis of bread with cricket flour.pdf]

Bread quality evaluation

Sensory analysis of bread was carried out according to the ISO 6658 method (2017) for overall acceptability by ten selected assessors using a ten-point Likert scale ranging from 1 (extremely dislike) to 10 (extremely like) [1]. Ten selected assessors from the internal recruitment (Department of Food Safety and Quality, Lithuanian University of Health Sciences, Lithuania, Kaunas) were chosen [2,3] (ISO 8586:2012; ISO 5492:2008). The previous training of the assessors was based on the descriptive analysis [4–6]. Selected assessors were no smoking persons, interested in sensory analysis and motivated.

References

1. ISO 6658 *Sensory Analysis—Methodology—General Guidance*; International Organization for Standardization Geneva, 2005;
2. ISO 8586 Sensory Analysis — General Guidelines for the Selection, Training and Monitoring of Selected Assessors and Expert Sensory Assessors Available online: https://www.iso.org/cms/render/live/en/sites/isoorg/contents/data/standard/04/53/45352.html (accessed on 11 August 2022).
3. Standardization, I.O. for ISO 5492: 2008. *Sens. Anal.* **2008**.
4. Torres, F.R.; Esmerino, E.A.; Carr, B.T.; Ferrão, L.L.; Granato, D.; Pimentel, T.C.; Bolini, H.M.A.; Freitas, M.Q.; Cruz, A.G. Rapid Consumer-Based Sensory Characterization of Requeijão Cremoso, a Spreadable Processed Cheese: Performance of New Statistical Approaches to Evaluate Check-All-That-Apply Data. *J. Dairy Sci.* **2017**, *100*, 6100–6110, doi:10.3168/jds.2016-12516.
5. Gaze, L.V.; Oliveira, B.R.; Ferrao, L.L.; Granato, D.; Cavalcanti, R.N.; Conte Júnior, C.A.; Cruz, A.G.; Freitas, M.Q. Preference Mapping of Dulce de Leche Commercialized in Brazilian Markets. *J. Dairy Sci.* **2015**, *98*, 1443–1454, doi:10.3168/jds.2014-8470.
6. Janiaski, D.R.; Pimentel, T.C.; Cruz, A.G.; Prudencio, S.H. Strawberry-Flavored Yogurts and Whey Beverages: What Is the Sensory Profile of the Ideal Product? *J. Dairy Sci.* **2016**, *99*, 5273–5283, doi:10.3168/jds.2015-10097.
