# Supplementary material for: Crickets (Acheta domesticus) as Wheat Bread Ingredient: Influence on Bread Quality and Safety Characteristics
Source: Foods. 2023 Jan 9;12(2):325. doi: 10.3390/foods12020325 (PMC9858247; doi:10.3390/foods12020325)
Supplement: Supplementary file 1 [file foods-12-00325-s001.zip › Supplementary File S5. Analysis of acrylamide concentration in bread .pdf]

Analysis of acrylamide concentration in bread

The acrylamide concentration was determined according to the method of Zhang et al. [24] with modifications, which are described below.

The bread samples were homogenized in blender (Ika A10, Germany). 2 g of sample were weighed in 50 mL centrifuge tube and diluted with 20 mL of distilled/deionized water. Tube was vortexed (ZX3 Advanced VELP, Italy) briefly to mix the contents of tube for 10 min. The tube was centrifuged at 4,000 rpm for 10 min with a centrifuge (Hermle Z 306, Germany).

The 10 mL of the clarified aqueous layer solution in 15 mL centrifuge tubes was clarified with 100 µl Carrez I (85 mM K4[Fe(CN)6] × $3H_2O$) and 100 µl Carrez II (250 mM ZnSO4×7H2O) solutions. The tubes were centrifuged at 4,000 rpm for 10 min. Acrylamide standard solution (30,4 µg/L). 15.2 mg of acrylamide analytical standard (99.8% purity) was weighed and dissolved in a 1000 mL volumetric flask and diluted with deionized water.

The obtained solution was diluted by pouring 2 mL of the obtained acrylamide solution into a 1000 mL measuring flask and diluted with deionized water. 3 mL of the sample supernatant (or standard solution) was derivatized in a glass tube by adding 1.5 g of potassium bromide (KBr), 1 mL of potassium bromate solution (0.1 M, $KBrO_3$) and 0.3 mL of sulfuric acid solution (50 %, H_2SO_4). The mixture was mixed in a shaker and kept for 2 h in a refrigerator (~4°C).

The derivative was neutralized by adding 250 µL of sodium thiosulphate solution (1 M, $Na_2S_2O_3x5H_2O$) until the orange colour disappears. About 1.5 g of sodium chloride (NaCl) was added to the derivatization mixture and the mixture was extracted with ethyl acetate ($CH_3COOC_2H_5$) (2 x 5 mL).

The collected ethyl acetate was concentrated with a concentration system (Christ CT 02-50, Germany) at a temperature of 40°C and reduced pressure. The solvent was evaporated and dissolved in 0.5 mL of ethyl acetate (for the standard, in a volume of 3 mL). The 100 mg of anhydrous sodium sulphate (Na_2SO_4), 20 µL of triethylamine ($(C_2H_5)_3N$) (20 µL of triethylamine in 0.5 mL of a concentrated derivatization solution) was added to the solution in 15 mL centrifuge tube, mixed and centrifuged for 10 minutes (4000 rpm).

The supernatant was analyzed by GC–ECD.A gas chromatograph (Shimadzu GC-17A, Japan) was equipped with an electron capture detector (ECD) and an integrator to measure peak areas, and a thermostatted column. Capillary column such as Rxi-5Sil MS (Restek, Germany): length 30 m; inner diameter 0.25 mm; stationary phase film thickness 0.25 µm. Working conditions: injection volume 1 µL; column temperature gradient 70°C (hold 1 min), 3°C/min to 140 (hold 0.5 min), 15°C/min to 280 (hold 4 min). Mobile phase nitrogen 18.0 cm/sec flow rate, split 3.0; injector temperature 250°C, detector temperature 260°C, detector current 2 nA.
