# Supplementary material for: Crickets (Acheta domesticus) as Wheat Bread Ingredient: Influence on Bread Quality and Safety Characteristics
Source: Foods. 2023 Jan 9;12(2):325. doi: 10.3390/foods12020325 (PMC9858247; doi:10.3390/foods12020325)
Supplement: Supplementary file 1 [file foods-12-00325-s001.zip › Supplementary File S6. Edible cricket flour volatile compounds.pdf]

Table S1. Non-treated and fermented edible cricket flour volatile compounds (volatile compounds, which in content in crickets flour samples was <1%).

RT, min	Volatile Compounds	Edible cricket flour		
		CoCr	CoCr	
		**Duration of fermentation, h**		
		**0**	**24**	**48**
10.235	3-hydroxybutanoic acid ethyl ester	nd	0.427 ± 0.065	nd
12.927	Acetic acid hexyl ester	0.879 ± 0.131 b	0.299 ± 0.059 a	0.264 ± 0.03 a
15.225	(4E)-4-hexen-1-ol acetate	nd	nd	0.442 ± 0.054
15.79	Undecane	0.589 ± 0.111 c	0.070 ± 0.011 a	0.304 ± 0.025 b
17.726	2,3,5-Trimethyl-6-ethylpyrazine	nd	0.183 ± 0.033 a	0.902 ± 0.073 b
17.845	5-(2-methylpropyl)-nonane	0.515 ± 0.038 b	0.150 ± 0.029 a	0.176 ± 0.025 a
18.757	2-Decanone	0.710 ± 0.098 b	0.236 ± 0.03 a	0.245 ± 0.023 a
19.135	(E)-2-Decen-1-ol	0.459 ± 0.063 b	0.251 ± 0.033 a	0.260 ± 0.034 a
19.295	4,4-dimethylundecane	nd	nd	0.076 ± 0.008
19.397	2,5-dimethylundecane	0.619 ± 0.077 c	0.163 ± 0.025 a	0.265 ± 0.027 b
19.641	4-methyldodecane	0.344 ± 0.061 c	0.118 ± 0.018 a	0.174 ± 0.030 b
20.28	1-(2-butoxy-1-methylethoxy)-2-propanol	nd	0.228 ± 0.044	nd
20.66	1,3-bis(1,1-dimethylethyl)-benzene	0.782 ± 0.071 c	0.281 ± 0.057 a	0.503 ± 0.097 b
21.051	Nonanoic acid	0.401 ± 0.066 b	0.593 ± 0.071 c	0.203 ± 0.043 a
21.311	(Z)-3-Octen-1-ol acetate	nd	nd	0.936 ± 0.209
21.39	2,6,10-trimethyldodecane	nd	0.297 ± 0.026 a	0.480 ± 0.077 b
21.565	tetrahydro-6-pentyl-2H-pyran-2-one	nd	nd	0.167 ± 0.017
21.789	2-Undecanone	nd	nd	0.633 ± 0.06
23.518	Propanoic acid, 2-methyl-, 2,2-dimethyl-1-(2-hydroxy-1-methylethyl)propyl ester	0.789 ± 0.151 b	0.309 ± 0.043 a	0.293 ± 0.042 a

Co – control; Cr – edible crickets; 122 – edible crickets fermented with *Lactiplantibacillus plantarum* No. 122 strain. The data expressed as mean values (n = 3) ± SE; SE – standard error. a–c Mean values between samples within a row with different letters are significantly different ($p \leq 0.05$).

Table S2. Correlations between fatty acids and volatile compounds content.

Volatile compound	r and p values	Fatty acid					
		C16:0	C16:1	C18:0	C18:1 *cis, trans*	C18:2	C18:3 α
Acetic acid	r	-0.023	-0.947**	0.168	-0.346	-0.401	0.156
	p	0.953	0.0001	0.667	0.361	0.285	0.689
Acetoin	r	0.380	-0.392	0.257	0.599	0.535	0.303
	p	0.313	0.297	0.504	0.088	0.138	0.428
2,3-Butanediol	r	0.384	-0.396	0.260	0.606	0.541	0.303
	p	0.308	0.291	0.499	0.084	0.133	0.427
Hexanal	r	0.565	-0.034	0.183	0.880**	0.843**	0.285
	p	0.113	0.930	0.638	0.002	0.004	0.457
Butanoic acid	r	-0.058	-0.497	0.274	-0.623	-0.627	0.391
	p	0.883	0.174	0.476	0.073	0.070	0.298
3-methylbutanoic acid	r	0.422	-0.555	0.318	-0.328	-0.354	0.432
	p	0.258	0.121	0.404	0.389	0.351	0.245
2-methylbutanoic acid	r	0.557	-0.642	0.397	0.318	0.250	0.496
	p	0.119	0.062	0.291	0.405	0.517	0.175
1-Hexanol	r	0.377	-0.389	0.256	0.594	0.531	0.302
	p	0.317	0.301	0.507	0.092	0.142	0.430
2-Heptanone	r	0.598	-0.516	0.437	-0.025	-0.065	0.559
	p	0.089	0.155	0.239	0.948	0.869	0.117
2,6-dimethylpyrazine	r	0.321	-0.445	0.251	-0.451	-0.461	0.352
	p	0.400	0.230	0.515	0.223	0.211	0.353
2,2-Dimethyl-3-heptanone	r	-0.007	-0.961**	0.070	-.0190	-0.255	0.038
	p	0.985	0.0001	0.857	0.624	0.509	0.922
2,6-dimethyl-4-heptanol	r	0.538	-0.544	.399	.462	.394	.499
	p	0.135	0.130	0.287	0.211	0.295	0.171
2-hydroxy-3-methylpentanoic acid methyl ester	r	-0.018	-0.972**	0.119	-0.299	-0.358	0.114
	p	0.963	0.0001	0.760	0.435	0.344	0.770
Benzaldehyde	r	0.395	-0.480	0.308	0.555	0.487	0.367
	p	0.292	0.191	0.420	0.121	0.184	0.331
1-Octen-3-ol	r	0.378	0.730*	-0.255	0.502	0.568	-0.061
	p	0.315	0.026	0.508	0.169	0.110	0.877
Phenol	r	0.278	-0.399	0.220	-0.500	-0.504	0.315
	p	0.469	0.288	0.569	0.170	0.167	0.409
2-pentylfuran	r	0.820**	0.480	0.042	0.690*	0.728*	0.288
	p	0.007	0.191	0.914	0.040	0.026	0.452
Decane	r	0.604	-0.229	0.275	0.811**	0.762*	0.423
	p	0.085	0.553	0.474	0.008	0.017	0.257
4-methyldecane	r	0.533	0.623	-0.185	0.640	0.693*	-0.107
	p	0.139	0.073	0.634	0.063	0.039	0.784
Benzeneacetaldehyde	r	-0.037	-.0915**	0.196	-0.468	-0.514	0.238
	p	0.925	0.001	0.614	0.204	0.157	0.538
3,6-dimethyldecane	r	0.254	0.745*	-0.380	0.487	0.553	-0.286
	p	0.509	0.021	0.314	0.184	0.122	0.455
3-ethyl-2,5-dimethylpyrazine	r	0.321	0-.657	0.166	-0.392	-0.422	0.210
	p	0.399	0.054	0.669	0.296	0.258	0.587
Tetramethylpyrazine	r	0.397	0.142	0.189	-0.290	-0.263	0.231
	p	0.290	0.715	0.626	0.450	0.494	0.550
2-Nonanone	r	0.408	-0.259	0.173	-0.370	-0.360	0.302
	p	0.276	0.502	0.657	0.328	0.341	0.430
5-methylundecane	r	0.285	0.683*	-0.092	0.372	0.439	0.047
	p	0.458	0.043	0.815	0.324	0.237	0.904
Nonanal	r	0.041	-0.498	-0.165	0.342	0.285	-0.297
	p	0.917	0.173	0.671	0.368	0.458	0.437
Phenylethyl Alcohol	r	0.041	-0.491	-0.162	0.338	0.281	-0.288
	p	0.917	0.180	0.677	0.374	0.464	0.452
3-methylundecane	r	0.370	0.696*	-0.291	0.534	0.598	-0.130
	p	0.328	0.037	0.447	0.138	0.089	0.739
Ethyl octanoate	r	0.365	0.505	-0.302	0.414	0.474	-0.121
	p	0.334	0.165	0.429	0.268	0.198	0.757
Dodecane	r	0.644	-0.473	0.451	0.005	-0.033	0.583
	p	0.061	0.199	0.222	0.990	0.934	0.099

r- Pearson correlation; p – significance. * Correlation is significant at the 0.05 level (2-tailed). ** Correlation is significant at the 0.01 level (2-tailed).
